# Fahr’s syndrome: literature review of current evidence

**DOI:** 10.1186/1750-1172-8-156

**Published:** 2013-10-08

**Authors:** Shafaq Saleem, Hafiz Muhammad Aslam, Maheen Anwar, Shahzad Anwar, Maria Saleem, Anum Saleem, Muhammad Asim Khan Rehmani

**Affiliations:** 1 Department of Medicine, Dow Medical College, DUHS, Karachi, Sindh, Pakistan; 2 3rd year student of Dow Medical College, DUHS, Karachi, Sindh, Pakistan; 3 1st year student of Karachi Medical and Dental College, Karachi, Sindh, Pakistan; 4 Assistant professor of Neurosurgery, Civil Hospital, Dow Medical College, DUHS, Karachi, Sindh, Pakistan

## Abstract

Fahr’s disease or Fahr’s syndrome is a rare, neurological disorder characterized by abnormal calcified deposits in basal ganglia and cerebral cortex. Calcified deposits are made up of calcium carbonate and calcium phosphate, and are commonly located in the Basal Ganglia, Thalamus, Hippocampus, Cerebral cortex, Cerebellar Subcortical white matter and Dentate Nucleus. Molecular genetics of this disease haven’t been studied extensively; hence evidence at the molecular and genetic level is limited. Fahr’s disease commonly affects young to middle aged adults. Etiology of this syndrome does not identify a specific agent but associations with a number of conditions have been noted; most common of which are endocrine disorders, mitochondrial myopathies, dermatological abnormalities and infectious diseases. Clinical manifestations of this disease incorporate a wide variety of symptoms, ranging from neurological symptoms of extrapyramidal system to neuropsychiatric abnormalities of memory and concentration to movement disorders including Parkinsonism, chorea and tremors amongst others. Diagnostic criteria for this disease has been formulated after modifications from previous evidence and can be stated briefly, it consist of bilateral calcification of basal ganglia, progressive neurologic dysfunction, absence of biochemical abnormalities, absence of an infectious, traumatic or toxic cause and a significant family history. Imaging modalities for the diagnosis include CT, MRI, and plain radiography of skull. Other investigations include blood and urine testing for hematologic and biochemical indices. Disease is as yet incurable but management and treatment strategies mainly focus on symptomatic relief and eradication of causative factors; however certain evidence is present to suggest that early diagnosis and treatment can reverse the calcification process leading to complete recovery of mental functions. Families with a known history of Fahr’s disease should be counseled prior to conception so that the birth of affected babies can be prevented. This review was written with the aim to remark on the current substantial evidence surrounding this disease.

## Definition

Basal ganglia calcification is also known as Fahr’s disease or Fahr’s syndrome. It is a rare inherited or sporadic neurological disorder with a prevalence of <1/1,000,000 [1-3]. It was first described by German neurologist Karl Theodor Fahr in 1930 [4].It is characterized by abnormal deposition of calcium in areas of the brain that control movements including basal ganglia, thalamus, dentate nucleus, cerebral cortex, cerebellum, subcortical white matter, and hippocampus [5].Most cases present with extra pyramidal symptoms initially. Additionally, they may present with cerebellar dysfunction, speech difficulty, dementia and neuropsychiatric symptoms [6].

### Molecular genetics

Fahr’s disease is most commonly transmitted as an Autosomal Dominant trait; but it may also be passed on as an autosomal recessive trait or it may occur sporadically [1,7]. A Locus at 14q (IBGC1) has been suggested to be involved commonly. A second locus has been identified on chromosome 8 and a third on chromosome 2 [8-10]. A loss of function mutation in the gene encoding type III sodium dependent phosphate transporter 2(SLC20A2) located on chromosome 8 has also been reported as the molecular level to form the genetic basis for the pathophysiology of this disease [11-13].

### Age of onset

Fahr’s syndrome typically affects individuals in the 3^rd^ and 4^th^ decades of their lives [2,14].

### Diagnostic criteria

Diagnostic criteria of Fahr’s syndrome has been modified and derived from Moskowitz et al. 1971, Ellie et al. 1989, Manyam 2005 [3,15,16] and it can be stated as follows:

• Bilateral calcification of the basal ganglia visualized on neuroimaging. Other brain regions may also be observed.

• Progressive neurologic dysfunction, which generally includes a movement disorder and/or neuropsychiatric manifestations. Age of onset is typically in the fourth or fifth decade, although this dysfunction may also present in childhood.^*^

• Absence of biochemical abnormalities and somatic features suggestive of a mitochondrial or metabolic disease or other systemic disorder.

• Absence of an infectious, toxic, or traumatic cause.

• Family history consistent with autosomal dominant inheritance.

### Synonyms

Fahr’s disease, Fahr’s syndrome, idiopathic basal ganglia calcification, striopallidodentate calcification and calcinosis nucleorum.

### ORPHA number

ORPHA 1980.

### Differential diagnosis

Symmetrical calcification of the basal ganglia occurs in a variety of familial and non-familial conditions; hence it doesn’t necessarily direct us towards a definitive diagnosis of Fahr’s syndrome.

• Congenital or early onset finding along with intellectual disability or presence of systemic involvement should alert one to the possibility of alternative diagnosis.

• When latent Tetany and myopathic changes occur with changes in somatosensory, visual and brain stem auditory responses, then parathyroid dysfunction, mitochondrial disease or other disease associated with brain calcification may be considered.

• Basal ganglia calcification, discovered in infancy along with ophthalmologic abnormality should prompt the consideration of infectious disease [17].

• It is differentiated from calcified angiomas, infections, enchapalitides and Addision’s disease by its severity and characteristic distribution.

## Methodology

Available literature concerning Fahr syndrome was analyzed. Literature search was conducted by using the following databases: Pub med, Google Scholar and Google. Key words used for searching were Fahr syndrome, Fahr disease and bilateral basal ganglia calcification. Using the word “Fahr’s syndrome” in Pub med, 64 articles were found from 1954–2013, term “Fahr’s disease” turned up 218 articles from 1951–2013 and the word “bilateral basal ganglia calcification” unearthed 172 articles from 1951–2013. Clinical data and diagnostic information was collected from abstracts or full text articles. Only articles written in the English language and case reports which had a definitive diagnosis of Fahr syndrome were included. Articles irrelevant to our study objectives were excluded.

### Diagnostic methods

#### Computed tomography

It is the preferable method of localizing and assessing the extent of Cerebral Calcification. Most frequently affected area is the lenticular nucleus, especially the internal globus pallidus while Cerebellar gyri, brain stem, centrum semiovale, and subcortical white matter may also affected. Calcifications in the putamen, thalami, caudate, and dentate nuclei are also common. Occasionally, calcium deposits begin or predominate in regions outside the basal ganglia. Calcification seems to be progressive and gradual.

#### MRI (magnetic resonance imaging)

Calcified areas in basal ganglia give a low intensity signal on a T2 image and low or high intensity signals on a T1 weighted plane [18]. The cerebellar lesions are found to be more heterogeneous. There may be a chance of high intensity signals in both T1 and T2 images due to reactive gliosis or degenerating tissue within calcified areas [17,19].

#### Plain skull radiograph

The plain skull radiograph has been shown to be the imaging modality of diagnostic value. Calcifications appear as clusters of punctate densities symmetrically distributed above Sella Turica and lateral to the midline, while subcortical and cerebellar calcifications appear wavy [17].

## Testing strategy for primary familial basal calcification

### Proband

Definitive diagnosis of primary familial basal calcification is established by Proband. For individuals in whom a diagnosis of Primary familial basal calcification is being considered, other causes of brain calcification should be eliminated prior to pursuing genetic testing. Such evaluations are as follows:

• Blood and urinary analysis to evaluate the disorders of calcium metabolism and heavy metal intoxication

• Cerebrospinal fluid analysis to assess for an infectious etiology and autoimmune disease.

If no other primary cause for brain calcification is detected or if the family history is suggestive of autosomal dominant inheritance, molecular genetic testing should be considered.

Sequencing of SLC20A2 should be undertaken first. If no mutation is identified, deletion/duplication analysis of SLC20A2 can be deliberated.

If no identifiable mutation or deletion in SLC20A2 is found, consider sequence analysis of PDGFRB.

If molecular genetic testing of both SLC20A2 and PDGFRB does not identify a disease causing mutation or deletion, consider other genetic diseases associated with brain calcifications.

Predictive testing for at-risk asymptomatic adult family members may include brain CT scan to evaluate calcium deposits. Presence of calcium deposits in this situation may be predictive of disease even in the absence of an identifiable familial mutation. In at-risk asymptomatic adult family members without brain calcifications, predictive testing requires prior identification of the disease-causing mutation in the family. Prenatal diagnosis and preimplantation genetic diagnosis (PGD) for at-risk pregnancies require prior identification of the disease-causing mutation in the family [17].

### Other baseline testing

In Fahr’s syndrome, certain hematological and biochemical investigations need to be carry out.

• Serum concentrations of calcium, phosphorus, magnesium, alkaline phosphatase, calcitonin and PTH should be tested.

• The Elisworth Howard test, yielding a 10–20 fold increase in urinary cAMP excretion following stimulation with 200 μmoles of parathyroid hormone aids in diagnosis.

• CSF evaluation of bacteria, viruses and parasite should be conducted

• Levels of ALP, urinary cAMP, serum creatinine, osteocalcin, serum lactic acid at rest and after exercise should be obtained. There is also an indication to measure the levels of 1,25 hydroxy Vit D3 [20,21].

• Natural Killer cells (NK) should be assessed in all suspected cases of Fahr’s syndrome as they were found to be raised in patients of Fahr’s syndrome [22].

### Epidemiology

To this date, very few studies have determined the frequency of basal ganglia calcification. In one study, 7040 patients were examined with CT scans, out of which 72(10.02%) showed symmetrical intracranial calcifications [23]. In a prospective study, calcification was detected in 30 out of 1478 (2%) patients on CT scans [24]. In another study, a review of 6,348 CT scans took place, out of which 39 (0.49%) confirmed the diagnosis of Fahr’s syndrome [25].

### Pathogenesis

Pathological features, being similar in both adults and infants, are not affected by age. At the molecular level, calcification generally develops within the vessel wall and in the perivascular space, ultimately extending to the neuron. Due to defective iron transport and free radical production, tissue damage occurs which leads to the initiation of calcification. It occurs secondarily around a Nidus composed of mucopolysaccharide and related substances. Progressive basal ganglia mineralization tends to compress the vessel lumen, thus initiating a cycle of impaired blood flow, neural tissue injury and mineral deposition. Basal ganglia concretions are recognized as basophilic globules tracking the vessels of arteries, veins and capillaries. Electron microscopy also shows the evidence of connection between spherical and hemispherical bodies formed in the adventitia of blood vessel and surrounding glial cells while intima is usually preserved. Microscopy reveals perivascular granules lying in the region above midbrain. The Lateral Pallidum is found to be more calcified relative to the Medial Pallidum. However, in another study, researchers found a sub-group with isolated calcification confined to Medial Pallidum [23,26]. Mineral composition of the calcifications varies with anatomical site and their proximity to vasculature calcifications. It may be due to the abnormal metabolism of calcium and phosphorus while some reports tend to contradict this finding [18,27,28]. In a review of 4219 CT scans, it was deduced that most calcifications occur bilaterally and symmetrically while a few occur unilaterally and there was no abnormality in metabolism of calcium, denying the pathophysiologic significance of concurrent altered calcium metabolism [29]. Calcifications commonly occur in basal ganglia, thalamus, dentate nucleus, cerebral cortex, cerebellum subcortical white matter, and hippocampus [14,18]. Deposits are composed of mineral compounds like calcium phosphate and carbonate; glyconate, mucopolysacchride and metals including Iron, Copper, Magnesium, Zinc, Aluminum, Silver and Cobalt may also be found [30-32].

## Etiology of Fahr’s syndrome

### Endocrine disorder

Endocrine disorders, particularly parathyroid disturbances are most commonly associated with Fahr’s syndrome. These abnormalities include idiopathic hypoparathyroidism, secondary hypoparathyroidism, pseudohypoparathyroidism, pseudo-pseudohypoparathyroidism and hyperparathyroidism. Idiopathic hypoparathyroidism is an uncommon condition characterized by the absence, fatty replacement or atrophy of the parathyroid glands. Secondary Hypoparathyrodism occur post-thyroidectomy as a complication of surgery. Pseudohypoparathyroidism is a condition associated primarily with resistance to the parathyroid hormone and the term Pseudopseudohypoparathyroidism is used to describe a condition where the individual has the phenotypic appearance of Pseudohypoparathyroidism type 1a, but is biochemically normal [17,24,33-35]. In a literature review of 150 cases, it was found that 35(23.3%) had idiopathic hypoparathyrodism whereas 23 (15.3%) had post thyroidectomy or secondary hypoparathyrodism [36,37]. Reduced parathormone (in hypoparathyrodism) or reduced renal response to parathormone (pseudohypoparathyroidism) causes hyperphosphatemia and hypocalcaemia, which in turn promotes calcification. In another study, it was found that Fahr’s syndrome occurred in 20(21.5%) of 93 patients with idiopathic hypoparathyrodism and 26(42.6%) of 61 patients with pseudo hypoparathyrodism [30,38]. Hypoparathyrodism is either primary or secondary and usually presents in first six decades of life and can be clinically recognized by Cataract, Tetany, Seizures, Dysarthria, increased Intracranial Pressure, Papilledema, soft tissue calcification, Congestive Heart failure, Pernicious anemia, dry hair, Alopecia, dental dysplasia, carries and predisposition to Moniliases [17,24]. In a recent case report, results of the histopathological examination revealed that there were five types of calcium deposits within the walls of capillaries and small and medium-sized arteries from the intracerebral affected areas. The histopathology specimen of the thyroid and parathyroid gland showed chronic lymphocytic thyroiditis and fibro-adipose tissue respectively. The symmetrical intracerebral calcifications were consequential of the hypoparathyrodism [39].

Pseudo and Pseudo-pseudohypoparathyroidism are depicted by a phenotypic spectrum caused by mutations in RNA and they also tend to occur in the same family. Average age of onset of PHP is at eight to ten years, and its clinical manifestations are similar with hypoparathyrodism except that intellectual disability is slightly more common in pseudohypoparathyroidism. Affected individuals have features of Albright Osteodystrophy. In Pseudopseudohypoparathyroidism, patients exhibit similar features but they do not have any biochemical abnormality, as opposed to patients of Pseudohypoparathyroidism. The serum concentrations of calcium and phosphorus are normal and there is a normal response to PTH secretion [17,40].

### Kenny Caffey syndrome type 1

Fahr’s syndrome has been known to be associated with the Kenny Caffey Syndrome Type 1. Being caused by a mutation in the TBCE gene, this syndrome is characterized by the symptoms of growth delay, cortical thickening of long bones, hypocalcaemia, hypothyroidism, and calcification of basal ganglia [41].

### Vitamin disorder

Vitamin D is a known player in the process of calcium metabolism due to which a disturbance in its homeostasis has significant implications for patients with Fahr’s syndrome [24].

### Mitochondrial myopathy

Due to the association of mitochondrial myopathy with abnormality in Calcium metabolism and increment in serum lactic acid and CSF protein, it was predicted that Fahr syndrome is also associated with Myopathies. One of these, the Kearn- Sayre syndrome, which consists of the triad of external opthalmoplegia, pigmentary retinal degeneration and increased CSF protein, has also been found to be associated with Fahr syndrome. In a study of 24 subjects from six kindreds with MELAS (myopathy, encephalopathy, lactoacidosis and stroke like syndrome), results revealed that bilateral basal ganglia calcification is the commonest radiological finding in mitochondrial disease [42,43].

### Adult onset neurodegenarative conditions

#### Neurodegeneration with brain iron accumulation disease (NBIA)

It is a form of NBIA and it presents with dystonia which is progressive, basal ganglia calcification dysarthria, rigidity and pigmentary retinopathy [17].

#### Neuroferritinopathy

It is also a form of NBIA and it typically manifests as adult onset chorea, dystonia and subtle cognitive defect along with excess iron storage or cystic degeneration in the Putamen [17].

#### Polycystic lipomembrenous osteodysplasia with sclerosing leukoencephalopathy

It is characterized by fractures resulting from polycystic osseous lesion, frontal lobe syndrome and progressive senile dementia beginning in the 4^th^ decade while bilateral calcifications are visualized on CT imaging [17].

### Dermatological disorders

Bilateral basal ganglia calcifications are correlated with a dermatological lesion, namely, lipoid protienosis which reveals itself with the observable conditions of seizures, dementia, hyalonisis of skin and mucous membrane, alopecia, photosensitivity and dwarfism [30].

### Infectious disease

#### Intrauterine or perinatal

Infections by Toxoplasmosis, rubella, CMV, herpes may result in calcifications of basal ganglia and dentate nucleus [17].

## Inherited congenital or early onset syndromes

### Cockayne syndrome

#### CS TYPE I

It comprises of developmental delay, hearing and visual loss, CNS and PNS disabilities along with Intracranial calcification of basal ganglia [17,35].

#### CS TYPE II

It is a more severe form of the Cockayne Syndrome, known as “Congenital Cockayne Syndrome, Cerebro-Occulofascial syndrome or Pena Shokeir type II syndrome. It consists of growth failure with little or none post natal neurologic development, congenital cataract, eye anomalies, kyphosis and joint contracture [17,35].

### Aicardi-Goutières syndrome

It is an early onset encephalopathy characterized by physical and mental handicap with calcification of basal ganglia, particularly Putamen, Globus Pallidus and thalamus, leukodystrophy, cerebral atrophy, CSF leukocytosis, increased concentration of interferon alpha in CSF, progressive microcephaly, irritability, feeding problems, sterile pyrexia, and in 20-25% of individuals by generalized tonic clonic seizures [17].

### Tuberous sclerosis complex

It is a disorder affecting multiple systems including abnormalities of skin (Hypermelanotic, Macules, Fascial Angiofibromas, Shagreen Patches, Fibrous Fascial Plaques, Ungual Fibroma), brain (Cortical Tubers, Subependymal Nodules, seizures, Intellectual disability/development delay), kidney (Angiolipoma, cysts) and heart (rhabdomyoma and arrhythmia). Cerebral hamartomas may be calcified and they are mainly periventricular or subcortical [17].

### Brucellosis

In a study, 65 cases of brucellosis were studied, of which 9(13.8%) were shown to have CT detected basal ganglia calcification. Out of these, 5 had meningitis and 4 had psychiatric manifestations. Calcification was unilateral in 3 cases, bilateral and symmetric in 4 cases and bilateral but asymmetric in 2 [44].

### Coat’s disease

A case report of a patient with bilateral basal ganglia calcification was successful in providing evidence that coat’s disease is associated with bilateral basal ganglia calcification [45].

### Normal aging

Calcification of the basal ganglia is an incidental finding in about 0.3%-1.5% of brain CT scans, especially in elderly individuals (age therefore being the most common cause). Microscopic calcifications can be observed in the Globus Pallidus and dentate nucleus in up to 70% of autopsy series. These calcifications are generally confined to the Globus Pallidus and do not have associated clinical findings [46,47].

## All etiological factors were combined in Table 1

**Table 1:** Etiological manifestations of Fahr’s syndrome

**S.No**	**Etiologies**	
1	Endocrine disorders	Idiopathic hypoparathyrodism
		Secondary hypoparathyrodism,
		Pseudohypoparathyroidism
		
		Pseudo-pseudohypoparathyroidism
		Hyperparathyroidism
2	Adult onset neurodegenerative conditions	Neurodegeneration With Brain Iron Accumulation Disease
		Neuroferritinopathy
		Polycystic Lipomembranous Osteodysplasia With Sclerosing Leukoencephalopathy
3	Infectious disease	Intrauterine and Perinatal infections
		Cockayne Syndrome Type 1
		Cockayne syndrome Type 2
4	Inherited or early onset syndrome	Aicardi-Gouteres Syndrome
		Tuberous Sclerosis Complex
		Brucellosis
		Coat’s disease

### Clinical features

#### Neurological features

A variety of neurological signs and symptoms are associated with Fahr’s syndrome. In adults loss of consciousness and seizures have been reported with hypothyroid hypocalcaemia [48,49]. Tetany is present, but it is difficult to distinguish from occasional myoclonus of epileptic disorder. Also, spasticity, gait disorder, speech impairment, dementia, parkinsonism, chorea, tremors, dystonia, myoclonus and coma are present. Papilledema of intracranial hypertension, Pleocytosis of CSF, paroxysmal Choreothetosis and paroxysmal dystonic Choreoathetosis have also been reported [50].

In a survey of 72 patients with intracranial calcifications, Kazis reported that extrapyramidal symptoms were present in 15(20.8%) patients, while in another study the frequency of extrapyramidal symptoms was calculated to be 56% [23,25]. In a research conducted on 982 people of rural Bavarian origin, 1(5.9%) of 17 Parkinsonism subjects had Fahr’s syndrome. Neurological manifestations may be confined to one side of the body despite the presence of bilateral calcifications [51,52].

Konig reported that one half of Fahr’s syndrome patients have neurological symptoms, while another study by Kazis reported a figure of 33.3% that is, one thirds of the patients were found to have them [23,25].

These statistics suggest that the prevalence of neurological symptoms may vary in FS. It has been suggested that extent or side of the lesion have an effect on manifestation but when it comes to dementia and extrapyramidal symptoms, they become worse with more extensive calcification [53]. Clinical findings may correlate with site of calcification as Lopes-Villega reported that out of 18 Fahr’s syndrome patients, 2(11.1%) had parkinsonism (with Pallidal thalamic and Cerebellal dentate calcification), 4(22.2%) had transient ischemic attacks ( Pallidal and Putamen calcification), 1(5.6%) had dysarthria and orthostatic hypotension (caudate and putamen calcification). Lesions were reported to be located in Globus Pallidus in 16(88.9%), putamen in 3(16.7%), caudate in 2(11%), thalamus in 1(5.6%) and cerebellar dentate nucleus in 1(5.6%) out of 18 patients [54].

### Movement disorder

Movement disorders in Fahr syndrome unveil as a spectrum of symptoms including clumsiness, fatigability, unsteady gait, slow or slurred speech, dysarthria dysphagia, involuntary movements and muscle cramping [16,27,55,56]. Neurological evaluation reveals that parkinsonism is present in 57%, chorea 19%, tremors 8%, dystonia 8%, athetosis 5% and orofacial dyskinesia in 3% of patients [2].

### Neuropsychiatric symptoms

These range from mild difficulty with concentration and memory to changes in personality or behaviors to psychosis and dementia [8,28]. A rare form of frontotemporal dementia with neurofibrillary tangles and Fahr’s type calcification was observed in a 50 year old Japanese woman who showed up with progressive dementia but neither extrapyrimidal symptoms nor metabolic disorder were observed [57]. Three cases with unusual type of pre-senile dementia were studied; calcareous deposition of Fahr’s type was present in each of them. Signs and symptoms were established to be neither those of Alzheimer nor those of Pick’s disease [58]. Patients with extensive calcification don’t seem to have a higher proportion of neurological impairment (35.8%) relative to those who show limited calcifications (34.5), but patients with extensive calcification did exhibit a higher frequency of psychiatric disorders (50%) than limited ones (34.5%). In another case, a 50 year old female patient with unimpaired basic and higher motor function had grossly compromised memory and attentional function but she remained neurologically asymptomatic. Neuropsychiatric illness may be present in the form of Schizophrenia like Psychosis, Dementia, Depression, Apoplexia, Deterioration of intelligence, inability to make decisions and effective organic alterations [28,53,59-62]. In one case report it was reported that reduced glucose uptake in PET scan was not only confined to the Putamen and Globus Pallidus, but extended to involve the temporal and parietal cortices, bilaterally. These functional changes may correspond to the neuropsychological deficits observed, i.e. disturbed selective attention and cognitive flexibility, verbal perseverations, and declarative memory deficits. It has been demonstrated that functional changes may precede cerebral atrophy in Fahr's syndrome and may reflect deficits in functional circuits, which involve both the basal ganglia and the frontal, parietal, and temporal lobes [63]. Lam et al. described two cases of Fahr’s syndrome with frontal lobe symptoms. One of them initially developed frequent uncontrollable bursts of laughter and crying and later, dysarthric speech while the second patient presented with progressive changes in personality and behavior [64].

## All clinical features were combined in Table 2

**Table 2:** Clinical features of Fahr’s syndrome

**S. No**	**Clinical features**	
1	Neurological	Loss of consciousness
		Tetany
		Seizures
		Epileptic disorder
		Gait disorder
		Spasticity
		Speech impairment
		Dementia
		Myoclonus
		Coma
		Paroxysmal choreothetosis
		Dystonic choreoathetosis
		Papilledema of intracranial Hypertension
		Pleocytosis of CSF
2	Movement disorder	Clumsiness
		Fatigability
		Unsteady gait
		Involuntary movements and muscle cramping
3	Neuropsychiatric features	Psychosis
		Depression
		Apoplexia
		Deterioration of intelligence
		Inability to make decisions

### Management of Fahr’s syndrome

To this date, various treatments have been administered to Fahr’s patients in an attempt to achieve remission or at least stabilization. Several approaches based on diverse biological theories and small scale clinical experiences have been proposed. Pharmacological treatment should be used to improve anxiety, depression, and obsessive compulsive disorder and to alleviate dystonia. Oxybutynin is used for urinary incontinence and antiepileptics are used for seizures [17]. Early diagnosis and treatment of the cause may abate the calcification process as described in a case in which treatment of hypoparathyrodism led to reversal of mental retardation in a 3 year old [65]. Seizures and movement disorders in Fahr’s syndrome which are related to the parathyroid disorder can be resolved with the correction of phosphate and calcium levels for e.g. treatment with alpha hydroxy vitamin D3 and corticosteroids reversed neurological deficits [52,66]. Clonazepam and atypical antipsychotic also offer a distinct advantage in treating patients with Fahr’s syndrome [24].

Great caution is indicated when using Lithium because it can further increase the risk of seizures in patients with Fahr’s syndrome. Treatment strategies involving the use of Carbamazepine, Benzipenes and Barbiturates may exacerbate underlying gait disorders [24].

Due to the absence of controlled data, psychiatrist or neurologist must remain vigilant in the conventional use of antidepressant and anxiolytic agents, the side effects of which may be precipitated at very low threshold in patients of Fahr’s syndrome as compared to individuals without disease.

## Genetic counseling issues

### Family planning

Optimal time for determination of genetic risk is before pregnancy.

### Testing of at risk asymptomatic adult

Brain CT scan serves as a pre-symptomatic test in at risk individual but its not useful for predicting age of onset, severity, type of symptoms or rate of progression in an asymptomatic individual. At risk individuals should submit themselves for testing when required to make personal decisions regarding conception, financial matters and career planning. Testing includes a pre-test interview in which motives for requesting individual knowledge of Fahr’s syndrome, possible impact of positive and negative result and neurological status are assessed [17]. Informed consent (including the possibility of incidental findings on a CT scan and whether such findings will be discussed) should be procured and records kept confidential. Individuals with a positive test result need arrangements for long-term follow-up and evaluations.

### Testing at risk asymptomatic individual during childhood

Consensus holds that individuals younger than 18 years who are at risk but asymptomatic should not be tested. Principal arguments against testing are that such testing removes individual future autonomy, causes psychological harm to child by altering self image, disturbing parent child or sibling-sibling relationships, increases anxiety and guilt because no treatment is currently available and it is devoid of any immediate medical benefit [17].

### Future perspective

There have been many major advances in our understanding of Fahr’s syndrome, especially in terms of etiology, the breadth of the disease phenotype and diagnostic methods. There is no simple solution to this dilemma and controversy. Certainly more qualitative and quantitative data on the experience of families are needed. Their voice - while reflecting one aspect of the whole portrait - is crucial and vital. Secondly, international consensus on guidelines for care that includes all of the specialties involved in the care of children and elder people with Fahr’s syndrome is required. Thirdly, Continuation of the now ongoing dialogue on this topic by Neurologist, geneticists, Psychiatrist, families, and the appropriate care specialists is mandatory and welcomed.

### Future research should focus on

1. The pathophysiological mechanisms leading to Bilateral Calcification. Only rare data are available on the early events leading to bilateral calcification.

2. The development of safe and effective medical treatments. Currently available drugs are either poorly effective and/or have bad tolerance.

## Conclusion

Idiopathic basal ganglia calcification or Fahr’s syndrome is a rare neurological disorder that is passed on in families as an autosomal dominant trait. This disorder is associated with a variety of other diseases but no specific etiologic agent has been identified yet. Diagnosis requires the presence of certain clinical criteria that may confuse the diagnosis with other conditions. New treatment modalities need to be discovered and employed to minimize loss of functionality associated with the disease. Of even more importance is to emphasize on the significance of genetic counseling of known at risk parents before conception. Screening asymptomatic individuals has not been able to demonstrate immediate medical benefits in adults or young individuals. It is of significant use only in adults as it can help them in decision making in their personal life regarding matters of finance, marriage and career. Screening of young individuals is considered unnecessary as it can result in psychological harm without being medically beneficial.

## Competing interest

Authors declared they had no competing interest.

## Authors’ contribution

SS, HMA and MA did manusript drafting and SA, MS, AS and MAKR did critical reviewing of manuscript. All authors read and approved the final manuscript.
